# Which melanoma patient carries a BRAF-mutation? A comparison of predictive models

**DOI:** 10.18632/oncotarget.9143

**Published:** 2016-05-02

**Authors:** Thomas Eigentler, Zeinab Assi, Jessica C. Hassel, Lucie Heinzerling, Hans Starz, Mark Berneburg, Jürgen Bauer, Claus Garbe

**Affiliations:** ^1^ Department of Dermatology, Center for Dermato Oncology, University Medical Center Tübingen, Tübingen, Germany; ^2^ Department of Dermatology and National Center for Tumor Diseases, University Hospital Heidelberg, Heidelberg, Germany; ^3^ Department of Dermatology, University Hospital Erlangen, Erlangen, Germany; ^4^ Department of Dermatology and Allergology, Klinikum Augsburg, Augsburg, Germany; ^5^ Department of Dermatology, University of Regensburg, Regensburg, Germany

**Keywords:** advanced melanoma, BRAF, predictive models, binary logistic regression, classification and regression trees

## Abstract

**Background:**

In patients with advanced melanoma the detection of BRAF mutations is considered mandatory before the initiation of an expensive treatment with BRAF/MEK inhibitors. Sometimes it is difficult to perform such an analysis if archival tumor tissue is not available and fresh tissue has to be collected.

**Results:**

514 of 1170 patients (44%) carried a BRAF mutation. All models revealed age and histological subtype of melanoma as the two major predictive variables. Accuracy ranged from 0.65–0.71, being best in the random forest model. Sensitivity ranged 0.76–0.84, again best in the random forest model. Specificity was low in all models ranging 0.51–0.55.

**Methods:**

We collected the clinical data and mutational status of 1170 patients with advanced melanoma and established three different predictive models (binary logistic regression, classification and regression trees, and random forest) to forecast the BRAF status.

**Conclusions:**

Up to date statistical models are not able to predict BRAF mutations in an acceptable accuracy. The analysis of the mutational status by sequencing or immunohistochemistry must still be considered as standard of care.

## INTRODUCTION

Since the first description, BRAFV600-mutations were recognized as major driver mutations in tumor progression of melanoma patients. [1] Initially reported to be present in 66% of the patients the frequency of BRAF V600 mutations was validated in larger cohorts and declined to 41–50%. [1–3] With the development of specific BRAF inhibitors such vemurafenib and dabrafenib, the presence of a BRAFV600-mutation is nowadays considered as one major target for the treatment of patients suffering from advanced melanoma. Right now, the detection of such a mutation is mandatory before the initialization of an effective but also expensive treatment. The mutation profiling is routinely performed by extracting DNA from a paraffin-embedded tumor tissue block followed by sequencing either by the Sanger method, pyrosequencing or real time PCR. In the last years even an immunohistochemistry driven proof of the presence of a BRAF V600E mutation was established.

In this paper a comparison of binary logistic regression model, a classification and regression (CART) analysis [4] and a random forest model [5] to predict the presence of a targetable BRAF mutation is presented. Such a model might be helpful to prioritize sample acquisition by biopsy when a patient is diagnosed with metastatic melanoma and there is no tissue left from the original source for BRAF mutation testing. Even though, BRAF testing is neither expensive nor time consuming in most developed countries, in places with limited resources, such a model could also help to prioritize BRAF mutation testing.

## RESULTS

Data from 1170 patients was available. Patients’ characteristics are shown in Table 1. BRAF status was available for all patients. 514 patients (44%) carried a BRAF mutation and 656 (56%) patients were non-mutated/wild-type. In detail, in 380 patients a BRAF-V600E was detected, followed by 65 patients carrying a V600K mutation. 50 patients were classified as being positive for a BRAF mutation by the COBAS 4800^®^ RT-PCR system, which does not differentiate between the subtypes of BRAF mutations and does not recognize the so-called V600E2 mutation (a V600E mutation coded not by GAG but by the Codon GAA). V600E2 is a false notion and describes just that the V600E mutation was not detected by the COBAS system, and was later discovered by Sanger sequencing. Other mutations were detected in single cases. Details are presented in Supplementary Table 1.

**Table 1:** Patients’ characteristics

	*N*	%
**Sex**		
Male	676	57.8
Female	494	42.2
**Age** [mean (sd)]	57.04 (14.53)	
**Type of melanoma**		
Acrolentiginous	78	6.7
Lentigo maligna	19	1.6
Unknown primary	118	10.1
Melanoma on a nevus	7	0.6
Mucosal	48	4.1
Nodular	225	19.2
Not classifiable	75	6.4
Ocular	15	1.3
Other types	35	3.0
Superficial spreading	243	20.8
Unknown	307	26.2
**Tumour thickness** [mean (sd)]	3.62 (3.53)	
**Localisation**		
Back	212	18.1
Bottom	14	1.2
Face	67	5.7
Foot	76	6.5
Hand	12	1.0
Head, other than face	84	7.2
Lower abdomen	25	2.1
Lower arm	12	1.0
Lower leg	89	7.6
Mucosal	21	1.8
Neck	25	2.1
Outer genital region	10	0.9
Thorax, Upper abdomen	85	7.3
Upper arm including elbows	45	3.8
Upper leg (incl. knee)	70	6.0
Unknown	323	27.6
**Ulceration**		
Present	334	28.5
Non-present	346	29.6
Unknown	490	41.9
**Age at Stage IV disease** [mean (sd)]	60.56 (13.86)	
**BRAF-Mutation-Status**		
K601E	1	0.1
L597Q	1	0.1
L597R	1	0.1
L597S	1	0.1
positive, not specified	50	4.3
V600D	2	0.2
V600E	380	32.5
V600E2	5	0.4
V600G	1	0.1
V600K	65	5.6
V600M	1	0.1
V600R	6	0.5
Wild-type	656	56.1

**Figure 1:**
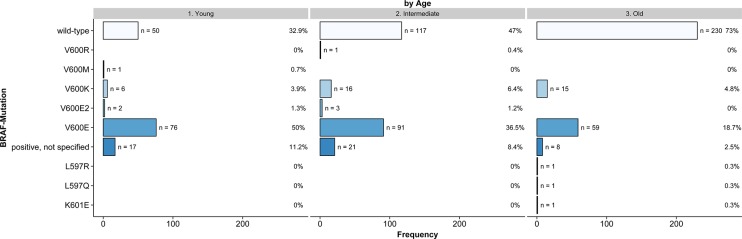
Frequency of BRAF mutations according to age (Young: < 45 Years, Intermediate: 45–59 Years, Old: ≥ 60 Years, *n* = 716, non-imputed)

Significant differences in the presence of BRAF mutations were calculated for age (*p* < 0.001, Figure 1), localization of the primary tumor (*p* < 0.001), tumor stage at initial diagnosis (*p* = 0.003), type of primary melanoma (*p* < 0.001) and tumor thickness (*p* = 0.005). Full details are presented in Table 2. Additionally, Supplementary Tables 1 and 2 illustrates the distribution of all variables according to the detected mutations.

**Table 2:** Contingency tables of difference variables and presence or absence of a BRAF-mutation, Fisher's exact testing for significance

	BRAF-Mutation			
	Missing	Mutated	Non-mutated	*p*
**n**	65	514	656	
**Age at entry stage IV disease** [mean (sd)]	59.87 (14.12)	51.73 (13.94)	61.31 (13.57)	< 0.001
**Sex** (%)				0.924
Male	36 (55.4)	298 (58.0)	378 (57.7)	
Female	29 (44.6)	216 (42.0)	278 (42.3)	
**Type of melanoma** (%)				< 0.001
Acrolentiginous	10 (15.4)	15 (2.9)	63 (9.6)	
Lentigo maligna	0 (0.0)	5 (1.0)	14 (2.1)	
Unknown primary	3 (4.6)	53 (10.3)	65 (9.9)	
Melanoma on a nevus	0 (0.0)	5 (1.0)	2 (0.3)	
Mucosal	11 (16.9)	2 (0.4)	46 (7.0)	
Nodular	3 (4.6)	113 (22.0)	112 (17.1)	
Not classifiable	4 (6.2)	42 (8.2)	33 (5.0)	
Ocular	1 (1.5)	3 (0.6)	12 (1.8)	
Other types	2 (3.1)	14 (2.7)	21 (3.2)	
Superficial spreading	13 (20.0)	135 (26.3)	108 (16.5)	
Unknown	18 (27.7)	127 (24.7)	180 (27.4)	
**Localisation** (%)				< 0.001
Acral	9 (16.4)	19 (4.7)	69 (15.0)	
Extremities	10 (18.2)	107 (26.5)	117 (25.4)	
Head/Neck	9 (16.4)	79 (19.6)	101 (21.9)	
Mucosal	12 (21.8)	0 (0.0)	20 (4.3)	
Trunk	15 (27.3)	199 (49.3)	154 (33.4)	
**Stage at initial diagnosis** (%)				< 0.001
I	8 (12.3)	63 (12.3)	47 (7.2)	
II	21 (32.3)	49 (9.5)	94 (14.3)	
III	6 (9.2)	92 (17.9)	107 (16.3)	
IV	8 (12.3)	34 (6.6)	34 (5.2)	
Unknown	22 (33.8)	276 (53.7)	374 (57.0)	
**TD** [mean (sd)]	3.38 (3.25)	3.24 (3.30)	3.94 (3.69)	0.019
**Ulceration** (%)				0.231
Non-present	16 (24.6)	165 (32.1)	181 (27.6)	
Present	24 (36.9)	136 (26.5)	198 (30.2)	
Unknown	25 (38.5)	213 (41.4)	277 (42.2)	

### Binary logistic regression model

**Figure 2:**
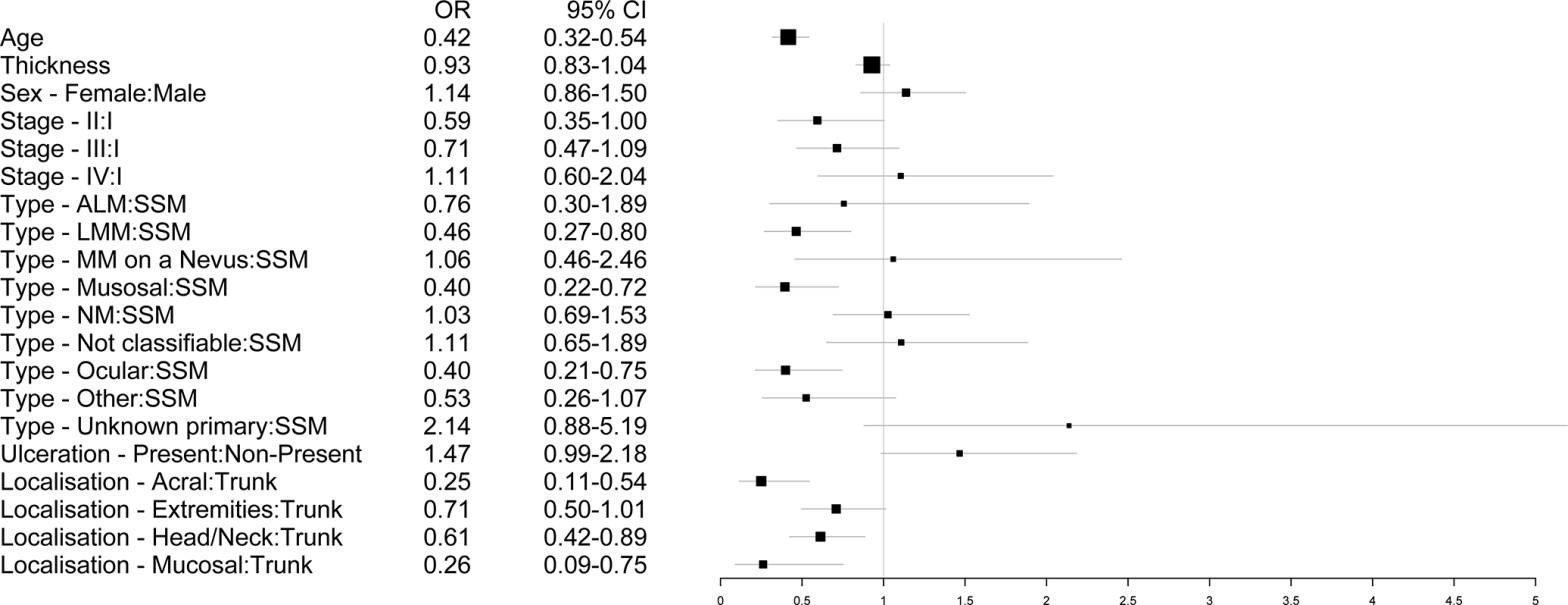
Forest plot illustrating the odd ratios with 95% confidence intervals of the different predictors for the binary regression model

**Figure 3:**
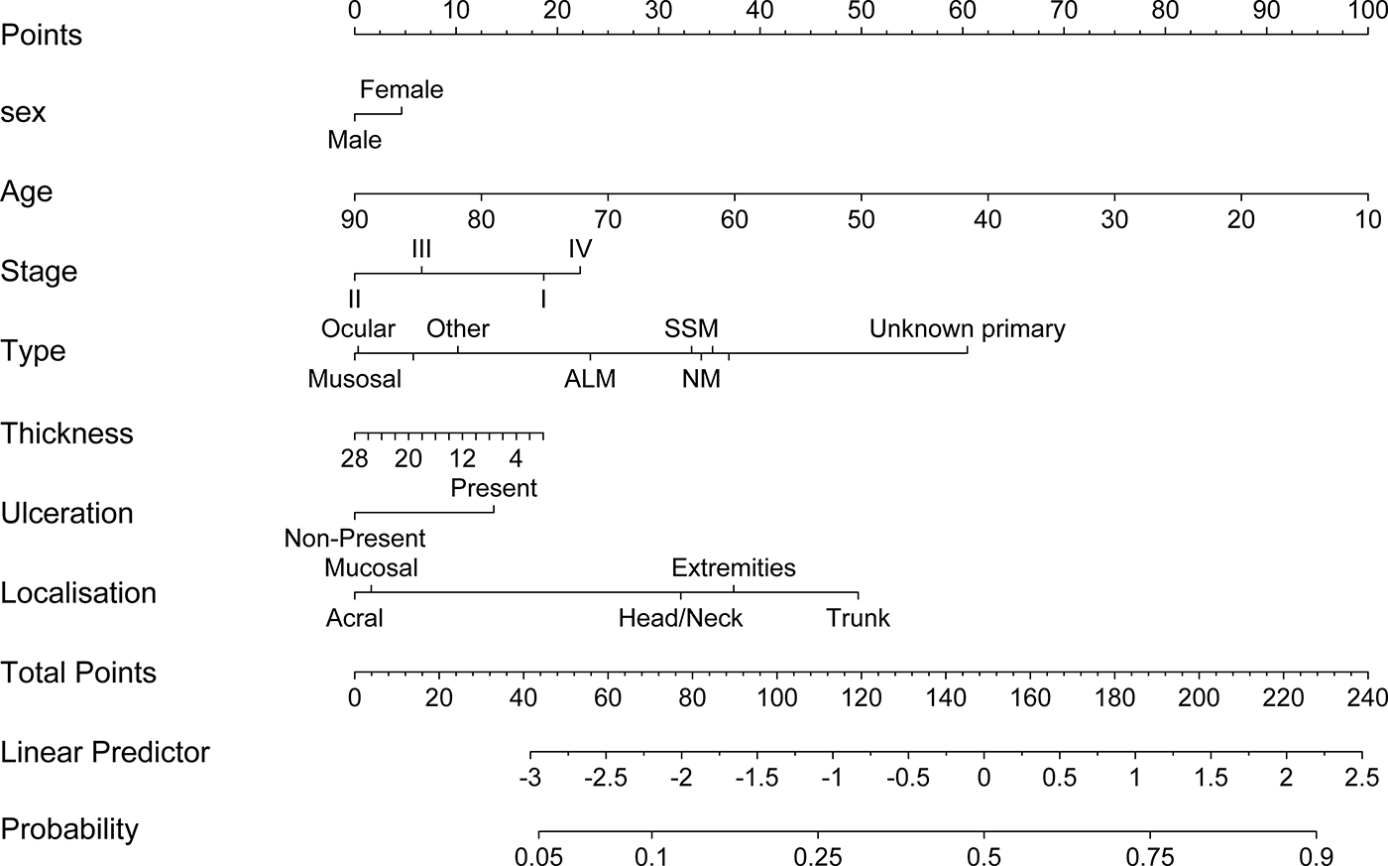
Nomogram predicting the presence of a BRAF mutation using a step-down model

The Wald-ANOVA calculation of our binary regression model revealed age (χ^2^ = 41.85, df = 1, *p* < 0.001), type of primary melanoma (χ^2^ = 38.68, df = 9, *p* < 0.001), localization of the primary melanoma (χ^2^ = 20.70, df = 4, *p* = 0.0004) and stage of disease at primary diagnosis (χ^2^ = 9.18, df = 3, *p* < 0.270) as significant predictive factors. The other factors such as gender (χ^2^ = 0.83, df = 1, *p* = 0.3626), thickness of the primary melanoma (χ^2^ = 1.75, df = 1, *p* = 0.1863), ulceration (χ^2^ = 3.57, df = 1, *p* = 0.0588) were non-significant. Figure 2 provides the corresponding forest plot of the odd ratios for the model, Supplementary Figure 1 the corresponding forest plot of the effects for the model. The accuracy of predicting the correct BRAF status was 0.6538 (95% CI: 0.6258–0.6811) with a sensitivity of 0.7683 and a specificity of 0.5078 (Table 3). In addition, a nomogram was calculated for our model (illustrated in Figure 3). An appropriate calibration plot is provided as Supplementary Figure 2.

**Table 3:** Comparison of different predictive models

Model	Accuracy	Accuracy (95% CI)	No Information Rate	Kappa	McNemar's Test *P*-Value	Sensitivity	Specificity
Binary logistic regression	0.6538	0.6258–0.6811	0.5607	0.2821	< 0.001	0.7683	0.5078
Classification and regression tree	0.6581	0.6301–0.6853	0.5607	0.2938	< 0.001	0.7576	0.5311
Random Forest	0.7171	0.6903–0.7428	0.5607	0.4099	< 0.001	0.8445	0.5545

### Classification and regression analyses

**Figure 4:**
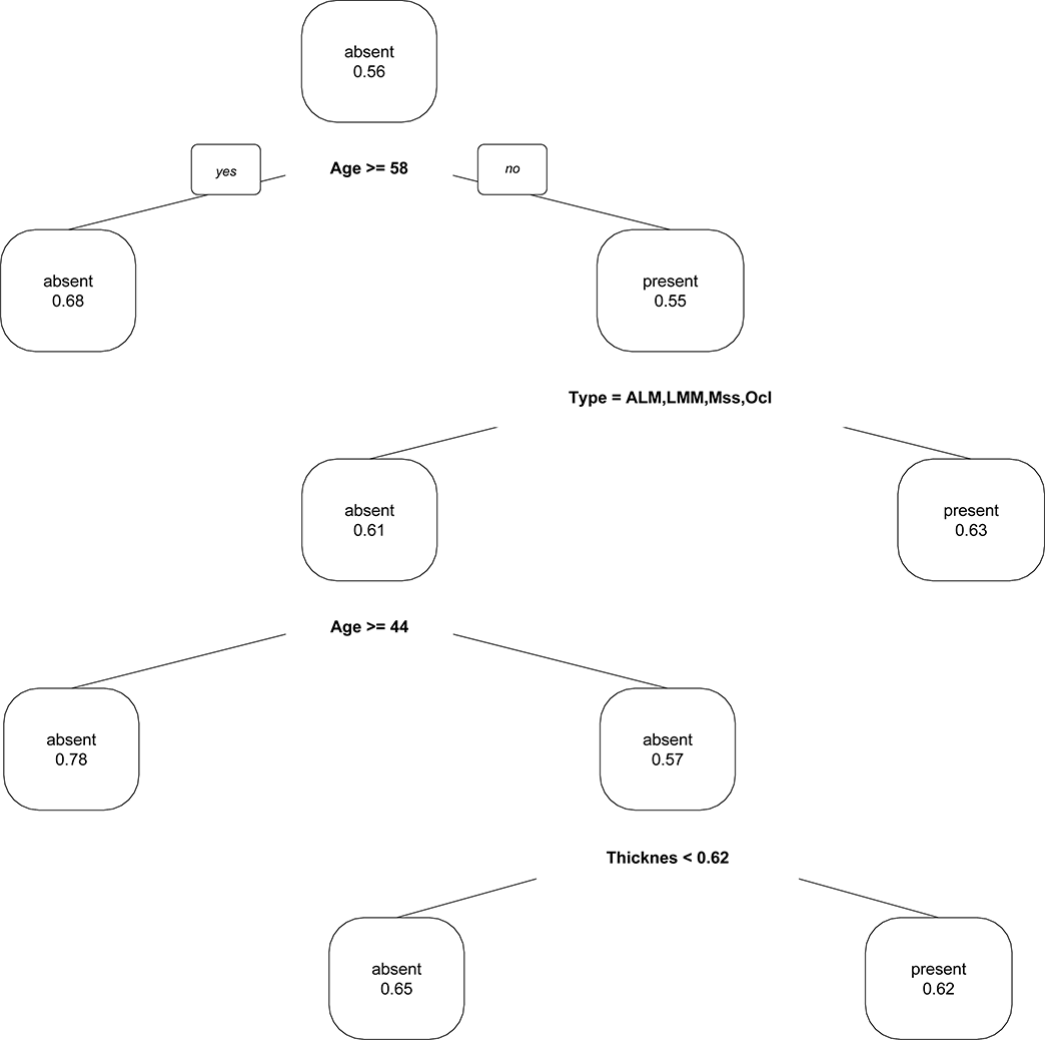
Classification and regression (CART) plot to predict the presence of a BRAF mutation

The decision tree of our CART analysis, trained on all 1170 cases, revealed the following structure: The first node splits at age ≥ 58 years, indicating that in patients of aged 58+ years the probability of carrying a BRAF mutation declines to 32%. The second node splits on the type of primary melanoma. Patients with a superficial spreading melanoma, nodular melanoma, melanoma on a nevus, with a melanoma which is not classifiable or of unknown primary have a probability carrying a BRAF mutation of 63%. The third node splits on age ≥ 44 years. Patients with an acrolentiginous melanoma, lentigo maligna melanoma, mucosal or an ocular melanoma being of age 44+ have only a probability of carrying a BRAF mutation of only 22%. The likelihood for patients being younger than 44 years and having a tumor thickness of less than 0.62 mm to carry a BRAF mutation is 35% whereas for patients with a melanoma with a thickness of 0.62 mm or above is 62%. A visualization of the tree is presented in Figure 4. The accuracy of predicting the correct BRAF status was 0.6581 (95% CI: 0.6301–0.6853) with a sensitivity of 0.7576 and a specificity of 0.5311 (Table 3).

### Random forest modelling

Finally, we performed a random forest model using the default set of 1000 trees, five candidate variables for each split with stopping criteria of at most observations within each terminal node. Supplementary Figure 3 illustrates a comparison of rankings of the minimal depth and variable importance. Both minimal depth and variable importance indicate a strong relation of age and type of melanoma variables to the forest prediction. The accuracy of predicting the correct BRAF status in this model was 0.7171 (95% CI: 0.6903–0.7428) with a sensitivity of 0.8445 and a specificity of 0.55545 (Table 3).

## DISCUSSION

We established three different prediction models for the presence or absence of a drugable BRAF mutation. All three models revealed age of the patients as well as subtype of the melanoma as the two major predictive variables. This confirms findings by Bauer and colleagues in 544 patients from Europe, USA and Australia. [6] For the European cohort they generated four different age groups (≤ 45 years, 46–60 years, 61–70 years and > 70 years). The main difference was calculated for the groups of patients aged 46–60 years and 61–70 years with a percentage of 60.6% vs 38.5% for the presence of a BRAF mutation. Interestingly, our classification and regression tree (CART) analysis revealed the age of 58 years as the optimal discrimination level.

Additionally, the histological subtype of melanoma is an important predictive variable confirmed in all three modeling approaches. This was also noted in a paper by Long et al. evaluating prognostic and clinical characteristics of 197 patients with advanced melanoma. [7] They detected that melanoma with the highest frequency of BRAF V600 mutations were superficial or nodular melanoma, whereas the probability of patients harboring a BRAF V600 mutation dramatically declined for acrolentiginous, ocular or mucosal melanoma. [7–9] Comparable findings were also published by Broekaert et al. [10] In their evaluation of 365 primary melanomas the majority of BRAF V600 mutations were detected in superficial spreading and nodular melanomas in non-chronically sun damaged localizations. A comprehensive overview of correlations of clinical and genetical features is provided in a review by Whitemen et al. [11].

Another important variable seems to be the localization of the primary tumor. In our binary regression model it was the third top ranked variable as it is in the ranking of the minimal depth and variable importance of our random forest model. In detail, the likelihood to carry a BRAF mutation is highest if the primary melanoma is located on the trunk. In acral location or in sun exposed areas such as the head and neck the odd ratio of carrying a BRAF mutated melanoma declines to 0.25 (acral location) and to 0.61 (head and neck region). Similarly, Bauer et al. reported about an independent association of BRAF mutation and localization of the primary melanoma. [6] In contrast, the histological subtype is closely correlated to the localization of the primary melanoma. So, this statistical independence is probably not clinically meaningful.

The initial stage of the disease also seems to be a variable that has to be taken into account. However, this variable was only relevant in our binary logistic regression model whereas tumor thickness was ranked higher in the random forest model. For both factors different prognostic impact values were reported in the literature. [7, 12].

The major question of this analysis was whether forecasting the BRAF status by modern predictive methods may be able to substitute molecular sequencing. This is obviously not the case. The accuracy of prediction was best in the random forest model with 0.71 and lowest in the binary regression model with 0.65. Whereas the sensitivity (truly predicting patients that carry a BRAF mutation) may be acceptable with 0.84 (random forest model) the specificity (truly predicting patients that do not carry a BRAF mutation) is unacceptable low with 0.55. In the latter case we would falsely classify patients as BRAF negative and refuse them an effective treatment.

In conclusion, this analysis confirmed known factors such as age and type of primary melanoma as variables correlating with the probability to carry a BRAF mutation. However, even with sophisticated statistical models we were not able to predict a BRAF mutation in a clinical acceptable range, so the analysis of the mutational status by sequencing or immunohistochemistry must still be considered as standard of care. This is even more important because the application of BRAF-inhibitors in BRAF wild-type patients can lead to a paradoxical MAP-kinase pathway activation resulting in an accelerated tumor growth and worse prognosis.

## MATERIALS AND METHODS

We collected the histological, mutational and clinical findings of patients from five major dermatological clinics specialized in the treatment of melanoma patients in Southern Germany. For the most part mutation detection was performed using pyrosequencing or conventional Sanger sequencing. Details about testing protocols are described elsewhere. [13–15] In selected cases mutational status was analyzed using a standardized real-time PCR (COBAS^®^ 4800) system provided by Roche. [15] The BRAF mutation was determined on the most recent available tumor tissue.

The following clinical variables were collected for the models: Sex, age at initial diagnosis, type of melanoma, tumor thickness according to Breslow, presence of ulceration, localization and the presence of a BRAF mutation. All variables were considered for the models. Potentially targetable BRAF-mutations (V600E, V600K, positive, not specified, V600R, V600E2, V600D, V600G, V600M) were selected as dependent variable. Missing values were imputed. The full population was used in development process of the models; the predictive performance of the models was validated by bootstrapping procedures with a number of 500 repetitions each. For the analysis of contingency tables Fisher's exact test was used to weight differences. For all tests values of 0.05 or lower were considered as significant.

All analyses were performed using R Version 3.1.1, [16] rms package version 4.4–0 [17] and caret package version 6.0–58. [18] Recalculation of random forest to provide graphical output data was performed using the randomForestSRC package version 1.6.1 [19] and plotting was done using function of the ggRandomForests package version 1.2.0. [20].

## SUPPLEMENTARY MATERIALS FIGURES AND TABLES


