# Supplementary material for: Which melanoma patient carries a BRAF-mutation? A comparison of predictive models
Source: Oncotarget. 2016 May 2;7(24):36130–7. doi: 10.18632/oncotarget.9143 (PMC5094988; doi:10.18632/oncotarget.9143)
Supplement: Supplementary file 1 [file oncotarget-07-36130-s001.pdf]

Which melanoma patient carries a BRAF-mutation? A comparison of predictive models

Supplementary Materials

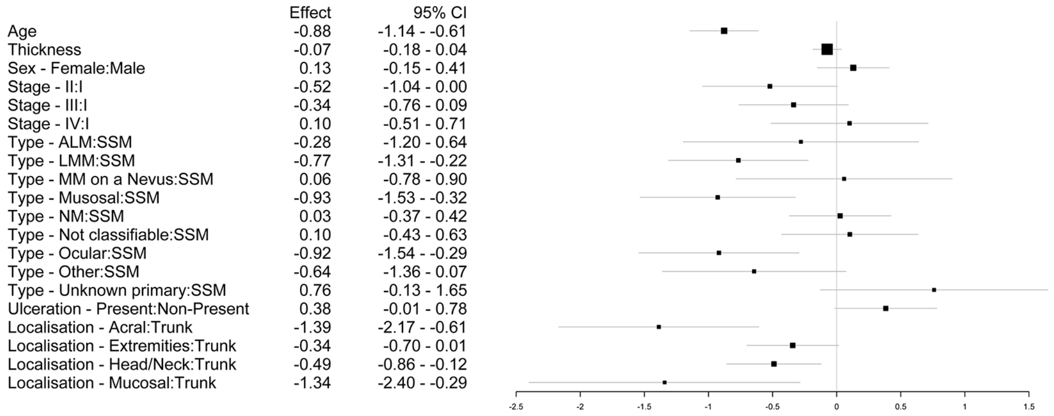

Supplementary Figure S1: Forest plot showing the effects with 95% confidence intervals of the different predictors for the binary regression model.

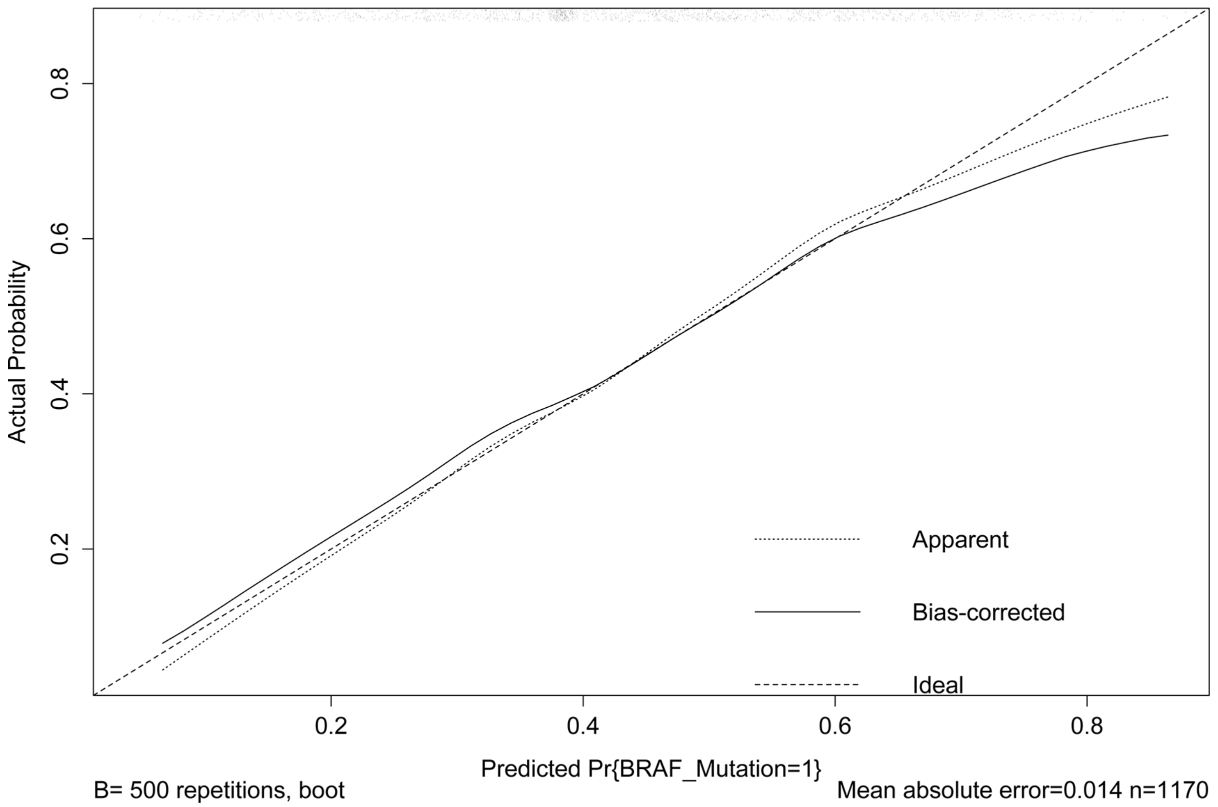

Supplementary Figure S2: Calibration plot for the binary regession model. Bootstap overfitting-corrected calibration curve estimate for the backwards step-down binary logistic model for presence of a BRAF-mutation, along with a rug plot showing the distribution of the predicted status. Loess smoothend nonparametric calibration estimator is used.

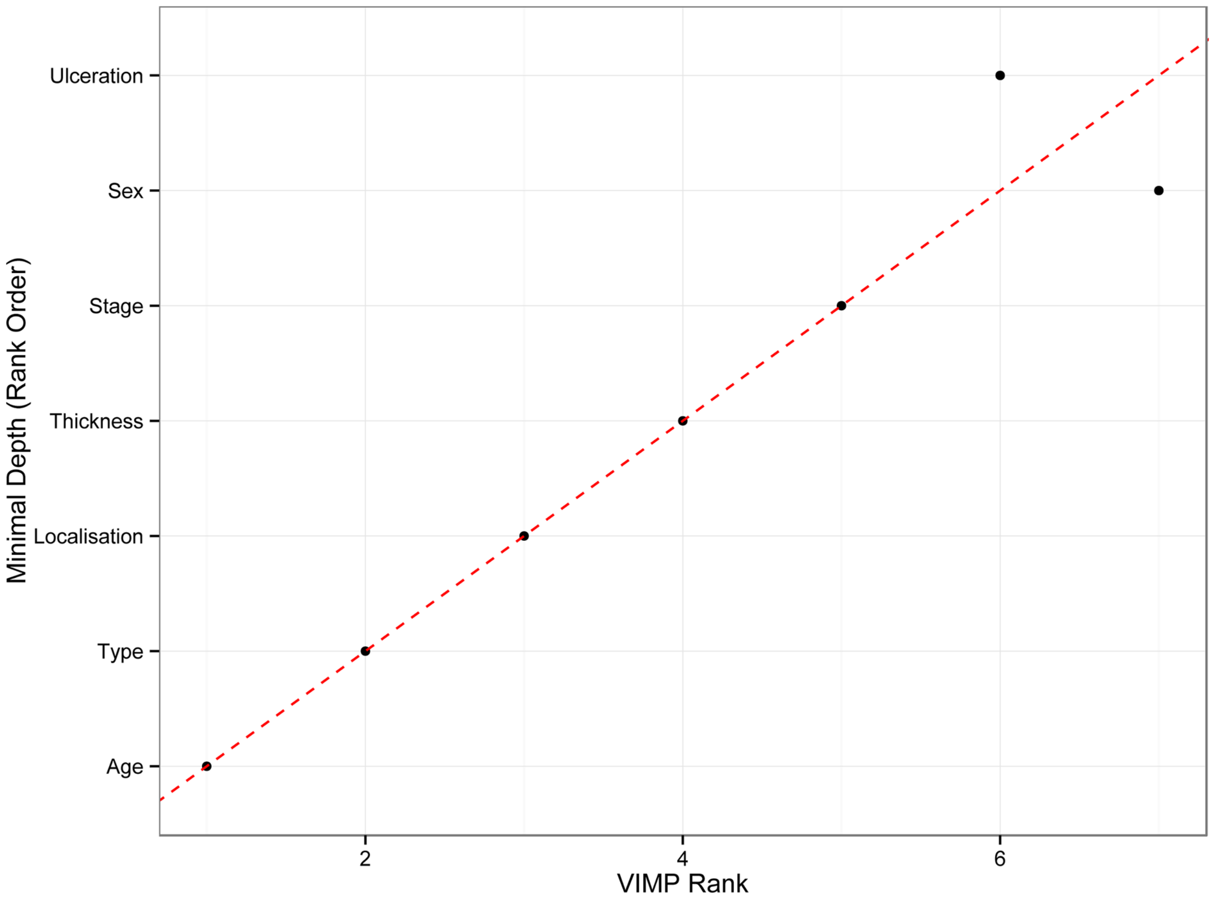

Supplementary Figure S3: Plot comparing minimal depth and variable importance (VIMP) rankings. Points on the red dashed line are ranked equivalently, points below have higher VIMP, those above have higher minimal depth ranking. Both minimal depth and VIMP indicate the strong relation of age and type of melanoma variables to the forest prediction.

Supplementary Table S1: Distribution of different variables according to detailed BRAF status

	Wild-type	V600E	V600K	Positive, not specified	V600R	V600E2	V600D	V600G	V600M	K601E	L597Q	L597R	L597S
N	656	380	65	50	6	5	2	1	1	1	1	1	1
**Sex** = Male (%)	378 (55.9)	210 (31.1)	44 (6.5)	31 (4.6)	4 (0.6)	3 (0.4)	1 (0.1)	1 (0.1)	1 (0.1)	1 (0.1)	1 (0.1)	0 (0.0)	1 (0.1)
**Sex** = Female (%)	278 (56.3)	170 (34.4)	21 (4.3)	19 (3.8)	2 (0.4)	2 (0.4)	1 (0.2)	0 (0.0)	0 (0.0)	0 (0.0)	0 (0.0)	1 (0.2)	0 (0.0)
**Age** (mean (sd))	61.31 (13.57)	50.85 (14.35)	58.97 (12.23)	50.07 (11.90)	55.00 (NA)	45.80 (7.50)			37.00 (NA)	74.00 (NA)	61.00 (NA)	67.00 (NA)	
**Localisation** (%)													
Acral	69 (78.4)	16 (18.2)	0 (0.0)	3 (3.4)	0 (0.0)	0 (0.0)	0 (0.0)		0 (0.0)	0 (0.0)	0 (0.0)		
Extremities	117 (52.2)	83 (37.1)	9 (4.0)	13 (5.8)	0 (0.0)	2 (0.9)	0 (0.0)		0 (0.0)	0 (0.0)	0 (0.0)		
Head/Neck	101 (56.1)	46 (25.6)	29 (16.1)	3 (1.7)	1 (0.6)	0 (0.0)	0 (0.0)		0 (0.0)	0 (0.0)	0 (0.0)		
Mucosal	20 (100.0)	0 (0.0)	0 (0.0)	0 (0.0)	0 (0.0)	0 (0.0)	0 (0.0)		0 (0.0)	0 (0.0)	0 (0.0)		
Trunk	154 (43.6)	149 (42.1)	18 (5.1)	22 (6.2)	3 (0.8)	3 (0.8)	1 (0.3)		1 (0.3)	1 (0.3)	1 (0.3)		
**Stage** (%)													
I	47 (42.7)	43 (39.1)	4 (3.6)	14 (12.7)	0 (0.0)	1 (0.9)			1 (0.9)	0 (0.0)			
II	94 (65.7)	31 (21.7)	7 (4.9)	8 (5.6)	1 (0.7)	1 (0.7)			0 (0.0)	1 (0.7)			
III	107 (53.8)	61 (30.7)	12 (6.0)	18 (9.0)	0 (0.0)	1 (0.5)			0 (0.0)	0 (0.0)			
IV	34 (50.0)	27 (39.8)	3 (4.4)	4 (5.9)	0 (0.0)	0 (0.0)			0 (0.0)	0 (0.0)			
**Type** (%)													
ALM	63 (80.7)	12 (15.4)	1 (1.3)	2 (2.6)	0 (0.0)	0 (0.0)	0 (0.0)	0 (0.0)	0 (0.0)	0 (0.0)	0 (0.0)	0 (0.0)	0 (0.0)
LMM	14 (73.7)	3 (15.8)	1 (5.2)	1 (5.2)	0 (0.0)	0 (0.0)	0 (0.0)	0 (0.0)	0 (0.0)	0 (0.0)	0 (0.0)	0 (0.0)	0 (0.0)
MM on a Ne-vus	2 (28.6)	5 (71.4)	0 (0.0)	0 (0.0)	0 (0.0)	0 (0.0)	0 (0.0)	0 (0.0)	0 (0.0)	0 (0.0)	0 (0.0)	0 (0.0)	0 (0.0)
Mucosal	46 (95.8)	0 (0.0)	0 (0.0)	1 (2.1)	0 (0.0)	0 (0.0)	0 (0.0)	0 (0.0)	0 (0.0)	0 (0.0)	0 (0.0)	1 (2.1.0)	0 (0.0)
NM	112 (49.8)	80 (35.7)	18 (8.0)	10 (4.4)	2 (0.9)	2 (0.9)	0 (0.0)	0 (0.0)	0 (0.0)	1 (0.4)	0 (0.0)	0 (0.0)	0 (0.0)
Not classifiable	33 (44.9)	35 (46.7)	4 (5.3)	2 (2.7)	0 (0.0)	1 (1.3)	0 (0.0)	0 (0.0)	0 (0.0)	0 (0.0)	0 (0.0)	0 (0.0)	0 (0.0)
Ocular	12 (80.0)	3 (20.0)	0 (0.0)	0 (0.0)	0 (0.0)	0 (0.0)	0 (0.0)	0 (0.0)	0 (0.0)	0 (0.0)	0 (0.0)	0 (0.0)	0 (0.0)
Other	21 (60.0)	13 (37.1)	1 (2.9)	0 (0.0)	0 (0.0)	0 (0.0)	0 (0.0)	0 (0.0)	0 (0.0)	0 (0.0)	0 (0.0)	0 (0.0)	0 (0.0)
SSM	108 (44.6)	93 (38.4)	15 (6.2)	20 (8.3)	1 (0.4)	2 (0.8)	0 (0.0)	1 (0.4)	1 (0.4)	0 (0.0)	1 (0.4)	0 (0.0)	1 (0.4)
Unknown primary	65 (55.1)	40 (33.9)	4 (3.4)	8 (6.8)	0 (0.0)	0 (0.0)	1 (0.8)	0 (0.0)	0 (0.0)	0 (0.0)	0 (0.0)	0 (0.0)	0 (0.0)

**Thickness** (mean (sd))	3.94 (3.69)	3.34 (3.56)	3.42 (2.93)	2.66 (2.14)	3.41 (1.58)	2.98 (2.99)		2.00 (NA)	0.50 (NA)	3.00 (NA)	2.10 (NA)	0.50 (NA)	0.85 (NA)
**Ulceration** = Present (%)	198 (59.3)	95 (28.4)	20 (6.0)	14 (4.2)	5 (1.5)	1 (0.3)	0 (0.0)	0 (0.0)	0 (0.0)	0 (0.0)	1 (0.3)	0 (0.0)	0 (0.0)
**Ulceration** = Ab-sent (%)	181 (52.3)	117 (33.8)	22 (6.4)	20 (5.8)	1 (0.3)	3 (0.9)	0	0	1 (0.3)	1 (0.3)	0	0	0

Supplementary Table S2: Distribution of different variables according to BRAF status (V600E, V600K and others; positive but not specified results were excluded)

	V600E	V600K	Others	*p*
N	380	65	19	
**Sex** = Male (%)	210 (55.3)	44 (67.7)	13 (68.4)	0.107
**Sex** = Female (%)	170 (44.7)	21 (32.3)	6 (31.6)	
**Age** (mean (sd))	50.85 (14.35)	58.97 (12.23)	52.30 (12.65)	0.005
**Localisation** (%)				
Acral	16 (4.2)	0 (0.0)	0 (0.0)	< 0.001
Extremities	83 (21.8)	9 (13.8)	2 (10.5)	
Head/Neck	46 (12.1)	29 (44.6)	1 (5.3)	
Mucosal	0 (0.0)	0 (0.0)	0 (0.0)	
Trunk	149 (39.2)	18 (27.7)	10 (52.6)	
Missing	86 (22.6)	9 (13.8)	6 (31.6)	
**Stage** (%)				
I	43 (11.3)	4 (6.2)	2 (10.5)	0.562
II	31 (8.2)	7 (10.8)	3 (15.8)	
III	61 (16.1)	12 (18.5)	1 (5.3)	
IV	27 (7.1)	3 (4.6)	0 (0.0)	
Missing	218 (57.4)	39 (60.0)	13 (68.4)	
**Type** (%)				
ALM	12 (3.2)	1 (1.5)	0 (0.0)	0.025
LMM	3 (0.8)	1 (1.5)	0 (0.0)	
MM on a Nevus	5 (1.3)	0 (0.0)	0 (0.0)	
Mucosal	0 (0.0)	0 (0.0)	1 (5.3)	
NM	80 (21.1)	18 (27.7)	5 (26.3)	
Not classifiable	35 (9.2)	4 (6.2)	1 (5.3)	
Ocular	3 (0.8)	0 (0.0)	0 (0.0)	
Other	13 (3.4)	1 (1.5)	0 (0.0)	
SSM	93 (24.5)	15 (23.1)	7 (36.8)	
Unknown primary	40 (10.5)	4 (6.2)	1 (5.3)	
Missing	96 (25.3)	21 (32.3)	4 (21.1)	
**Thickness** (mean (sd))	2.53 (1.95)	3.34 (3.56)	3.42 (2.93)	0.652
**Ulceration** = Present (%)	95 (25.0)	20 (30.8)	7 (36.8)	0.512
**Ulceration** = Absent (%)	117 (30.8)	22 (33.8)	6 (31.6)	
**Ulceration** = Unknown (%)	168 (44.2)	23 (35.4)	6 (31.6)	
